# Commentary: Functionality of Two Origins of Replication in *Vibrio cholerae* Strains With a Single Chromosome

**DOI:** 10.3389/fmicb.2019.01314

**Published:** 2019-06-19

**Authors:** Bhabatosh Das, Dhruba K. Chattoraj

**Affiliations:** ^1^Translational Health Science and Technology Institute, Faridabad, India; ^2^Center of Cancer Research (CCR), National Cancer Institute (NCI) and National Institute Health (NIH), Bethesda, MD, United States

**Keywords:** chromosome fusion, *V. cholerae* Chr2 replication, replication enhancer, CRTs, divided genome

This paper is about divided genomes in bacteria. In the era of genomics, it has become clear that about 10% of bacteria have multiple chromosomes, as is the norm in eukaryotes. This observation raises the questions as to how they originated and how they are maintained, in particular whether their replication and segregation are independently or coordinately controlled, and what evolutionary advantage the divided genome might have to discourage reversion to the single-chromosome state, the norm in bacteria.

The prevailing view is that multi-chromosome bacteria have originated from single-chromosome bacteria by transferring some essential genes from the chromosome to plasmids, thus making the plasmid an indispensable component of the genome or in other words, another chromosome (Fournes et al., 2018). The best evidence for this view comes from studies of *Vibrio cholerae* (Vc), which has one main chromosome (Chr1), analogous to the paradigmatic *Escherichia coli* chromosome, carrying most of the housekeeping genes, and a second chromosome (Chr2) with distinct hallmarks of certain low-copy number *E. coli* plasmids, such as P1 and F, but carrying some essential genes not present in Chr1.

Genomes of many naturally occurring *Vibrio* strains have been analyzed, and in the *Vibrionaceae* family that includes Vc, the two-chromosome genome has been the rule. However, in a recent analysis of 91 *Vibrio* strains from the Sakazaki collection, two strains were found with a single chromosome that resulted from fusion of Chr1 and Chr2 (Chapman et al., 2015; Xie et al., 2017). This is the first report of a naturally occurring single-chromosome *Vibrio* (NSCV), although forced fusions in the laboratory were achieved earlier (Val et al., 2012, 2014, 2016). Since then, another *Vibrio* with single chromosome has been reported (Yamamoto et al., 2018). Note that in all the laboratory-achieved fusions, the Chr2 replicon was inactive, and the strains survived because Chr2 could be passively maintained as an integral part of Chr1. In contrast, both Chr1 and Chr2 origins (*ori1* and *ori2*) were active in the strain NSCV1 of Xie et al. (Figure 1) (Bruhn et al., 2018). The commentary is based on this exceptional finding.

**Figure 1:**
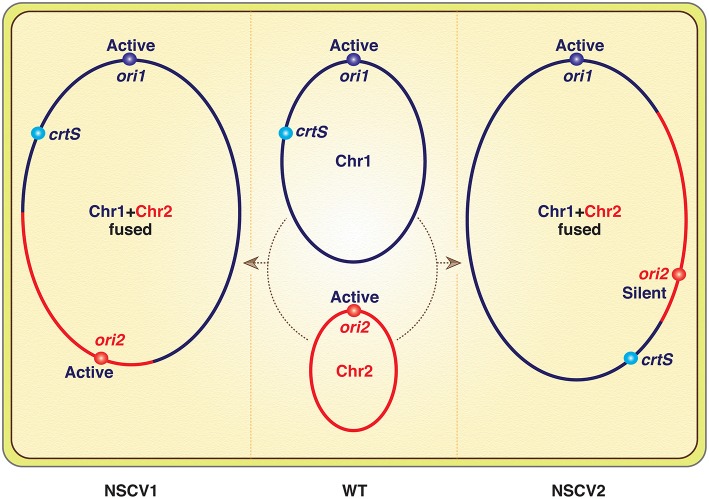
Schematics of *V. cholerae* chromosomes (Chr1 and Chr2) before (in WT) and after fusions (in NSCV1 and NSCV2). Note that a replication fork emanating from *ori1* will encounter *crtS* before *ori2* in NSCV1 whereas the opposite will be the case in NSCV2. The inactivity of *ori2* in NSCV2 is expected since *crtS* duplication is a prerequisite for *ori2* firing, although the authors have reasons to believe that this may not be the real explanation.

The basic claim that both the origins can function in a fused chromosome is reasonable. Particularly, the authors verified that the two special features of Chr2 replication, dependence on Dam methylation and on two copies of a replication enhancer site *crtS*, are retained after the fusion. In the other fused chromosome strain (NSCV2), the fusion junctions were different, and *ori2* was silent. The authors attributed this to an altered genomic context of the regulatory sites (*ori1, ori2*, and *crtS*, Figure 1) and not on their relative positions, which is currently believed to be important for *ori2* function. Although how the context matters was not elaborated on, a new perspective on Chr2 replication was provided to explain the results. The idea is that the regulation of Chr2 replication is such that it maintains the parity of *crtS* to *ori2* copy numbers (de Lemos Martins et al., 2018; Ramachandran et al., 2018). The *crtS* site normally resides in Chr1 and when the site number doubles upon passage of the Chr1 replication fork, Chr2 replication initiates and restores the *crtS*/*ori2* ratio.

A bacterial chromosome with two functional origins is unprecedented and is unexpected. A reason for why bacterial chromosomes have one origin whereas eukaryotic chromosomes have multiple origins, has been proposed (Kuzminov, 2014). In eukaryotes, chromosomes segregate at the end of replication, and the entire chromosome segregates as a unit, whereas in bacteria the two arms of a replication bubble start segregating away from each other soon after their synthesis. In other words, segregation proceeds much before the completion of replication. If there are two replication bubbles on the same chromosome from two differently located origins, then productive segregation of the replicated arms would require that the parental Watson strand of both the bubbles go in the same direction, and the parental Crick strand of both the bubbles go in the opposite direction. No mechanism for such non-random segregation is known. It might well be that to avoid random segregation of locally replicated arms, which can potentially entangle rather than segregate the replicated arms, bacteria with a single origin might have enjoyed a significant selective advantage.

The two single-chromosome strains, however, were stable when grown over 160 generations. How? Fusion junctions indicate that complex genetic rearrangements accompanied the joining of the two chromosomes, which would prevent the simple reversal of the integration event. As argued above, the stability of an irreversibly fused chromosome can be improved by silencing one of the origins, which is the case in NSCV2. In NSCV1, it is still possible that only one of the functional origins fires in any one cell cycle. Even if both the origins fire in the same cell cycle, then silencing or overriding of one of the two segregation systems of Vc would avoid the mess that random segregation of replicated arms might cause. Also, Chr1 initiates replication first and its segregation system is set in motion well-before the onset of the Chr2 segregation. In the fused chromosome, the Chr1 system most likely dominates, which is a testable prediction.

The finding that chromosomes can fuse and that the fused chromosome can be stably maintained with two functional origins raises the question: What keeps the chromosomes from fusing in the vast majority of cases? This is even more surprising because the chromosomes share plenty of regions for homologous recombination (Heidelberg et al., 2000). Fused-chromosome strains should be viewed as an exception and, moving forward, the emphasis should be on understanding the selective advantages of maintaining the divided state. Since the majority of bacteria have one chromosome, the selection of the divided state must have some species-specific basis. For example, *Vibrios* are one of the fastest growing bacteria (Lee et al., 2019). The high growth rate entails multi-fork replication and dividing the genome lessens the demand for more forks (Srivastava and Chattoraj, 2007). A chromosome with fewer forks should be less vulnerable to damage, which would be worth exploring.

## Author Contributions

All authors listed have made a substantial, direct and intellectual contribution to the work, and approved it for publication.

### Conflict of Interest Statement

The authors declare that the research was conducted in the absence of any commercial or financial relationships that could be construed as a potential conflict of interest.
